# Diagnostic advances in medullary thyroid carcinoma

**DOI:** 10.1530/EO-25-0053

**Published:** 2025-10-07

**Authors:** Petra Petranović Ovčariček, Luca Giovanella

**Affiliations:** ^1^ Department of Oncology and Nuclear Medicine, University Hospital Center Sestre Milosrdnice, Zagreb, Croatia; ^2^ Clinic for Nuclear Medicine and Thyroid Center, Gruppo Ospedaliero Moncucco, Lugano, Switzerland; ^3^ Clinic for Nuclear Medicine and Interdisciplinary Thyroid Center, University Hospital and University of Zurich, Zurich, Switzerland

**To the editor,**

We observed with great interest the recent article by Jara and Castroneves, ‘Overview of management and therapeutic advances in medullary thyroid cancer’, published in volume 5, part 1, Article ID e240077 (Jara & Castroneves 2025). The authors delivered an extensive and relevant summary of present approaches to diagnosing, treating, and monitoring medullary thyroid carcinoma (MTC). We recognize their significant contribution to this area of study. However, we would like to offer several constructive comments concerning the interpretation of serum calcitonin (CT) testing and the application of molecular imaging techniques, particularly positron emission tomography (PET), which may further enhance the diagnostic and therapeutic strategies in MTC.

## Calcitonin

The authors highlighted the limited availability of pentagastrin stimulation and the inter-assay variability of CT immunoassays as key limitations in the clinical utility of serum CT in diagnosing MTC. While we acknowledge these points, the stimulation test is no longer as relevant as it once was, and straightforward strategies are now available to enhance the accuracy of CT measurement. Calcium gluconate, introduced as an alternative CT secretagogue in 2009, has proven to be a reliable and safe stimulant (Doyle *et al.* 2009). However, accumulating evidence suggests that basal CT (bCT) measurement using modern high-sensitivity immunoassays offers diagnostic performance equal to or superior to that of stimulation tests. For instance, Niederle *et al.* compared bCT and calcium-stimulated CT (Ca-sCT) levels in 149 patients with thyroid nodules and elevated bCT, using a next-generation immunochemiluminometric assay. The diagnostic accuracy of bCT was superior in both males (AUC: bCT 0.894; Ca-sCT 0.849) and females (AUC: bCT 0.935; Ca-sCT 0.868), and the combination of both tests did not yield added value (Niederle *et al.* 2020). These findings were corroborated in a larger retrospective cohort study involving 12,984 patients (Broecker-Preuss *et al.* 2023).

Accordingly, current clinical guidelines have adopted bCT measurement as the preferred approach in both diagnostic work-up and follow-up of MTC, rendering stimulation testing largely obsolete, with the exception of selected inherited cases requiring precise surgical timing (Wells *et al.* 2015). Nevertheless, CT testing is susceptible to methodological and physiological confounders, necessitating thorough assay validation and interpretation (Bae *et al.* 2025). Importantly, new-generation assays demonstrate enhanced analytical performance, reduced inter-individual variability, and diminished influence of non-MTC-related factors; however, such problems are still far from infrequent in clinical practice, and inter-assay variability remains an unresolved issue.

## Procalcitonin as an alternative/complementary MTC biomarker

Procalcitonin (ProCT), a CT precursor with favourable analytical properties, has gained attention as a potential biomarker for MTC. Unlike CT, ProCT exhibits a stable and predictable half-life (20–24 h) and superior serum stability. Its expression in healthy individuals is restricted to thyroid C-cells, and several studies have shown excellent diagnostic accuracy in MTC, particularly in cases of spurious CT elevation or fluctuating post-operative CT levels. A recent meta-analysis reinforced the clinical utility of ProCT in MTC, suggesting its potential to complement or even replace CT in certain settings (Giovanella *et al.* 2021). At minimum, ProCT should be integrated into clinical protocols as an adjunct marker, especially in cases of indeterminate CT dynamics or in diagnostic algorithms aimed at ruling out MTC in patients with thyroid nodules.

## Molecular imaging in MTC

We concur with the authors on the pivotal role of molecular imaging in MTC, especially in detecting recurrent or metastatic disease. ^18^F-DOPA PET/CT is currently the most sensitive modality in patients with elevated CT levels (≥150 pg/mL), with reported patient-based sensitivity, specificity, and accuracy of 95, 93, and 94%, respectively (Giovanella *et al.* 2020). Nonetheless, other radiopharmaceuticals merit consideration. In advanced or dedifferentiated MTC, where carcinoembryonic antigen levels disproportionately exceed CT and tumour marker doubling times are reduced, ^18^F-FDG PET/CT may outperform ^18^F-DOPA in prognostication and identifying aggressive phenotypes. All in all, while ^18^F-DOPA remains the modality of choice for disease mapping, ^18^F-FDG is superior in identifying patients with biochemical progression and poorer outcomes. Furthermore, imaging with ^68^Ga-labelled somatostatin analogues (SSA) is gaining traction, particularly in cases where ^18^F-DOPA is not readily available. Notably, however, its diagnostic performance is inferior to either ^18^F-DOPA or ^18^F-FDG, with the possible exception of skeletal metastasis. Accordingly, using ^68^Ga-SSA PET/CT as an alternative to other radiopharmaceuticals remains debatable. On the other hand, ^68^Ga-SSA PET/CT may be valuable when therapeutic options such as ^177^Lu-DOTATATE peptide receptor radionuclide therapy (PRRT) are being considered in order to assess the feasibility of PRRT and predict its effectiveness (Hayes *et al.* 2021). Molecular imaging should be employed in selected cases (relapsing disease, advanced disease), and radiopharmaceuticals selected based on clinical and pathological characteristics, disease burden, serum biomarker levels, and kinetics (i.e. doubling time) (Giovanella *et al.* 2022).

## Conclusions

In conclusion, we congratulate the authors on their excellent synthesis of current MTC management strategies. We suggest that recent advancements in biomarker technology – particularly the integration of ProCT – and a tailored approach to molecular imaging with an appropriate selection of PET radiopharmaceuticals may further refine and personalise care for patients with MTC.

## Declaration of interest

The authors declare that there is no conflict of interest that could be perceived as prejudicing the impartiality of the work reported.

## Funding

This work did not receive any specific grant from any funding agency in the public, commercial, or not-for-profit sector.

## Editor's note

A response from Jara & Castroneves has been requested. This letter has been published in advance of any reply, in order not to delay publication of the potentially important points made in this letter.

## References

[bib1] Bae YJ, Schaab M & Kratzsch J 2025 Calcitonin as biomarker for the medullary thyroid carcinoma. Recent Results Cancer Res 223 155–182. (10.1007/978-3-031-80396-3_6) 40102257

[bib2] Broecker-Preuss M, Simon D, Fries M, et al. 2023 Update on calcitonin screening for medullary thyroid carcinoma and the results of a retrospective analysis of 12,984 patients with thyroid nodules. Cancers 15 2333. (10.3390/CANCERS15082333) 37190260 PMC10136489

[bib3] Doyle P, Düren C, Nerlich K, et al. 2009 Potency and tolerance of calcitonin stimulation with high-dose calcium versus pentagastrin in normal adults. J Clin Endocrinol Metab 94 2970–2974. (10.1210/JC.2008-2403) 19491231

[bib4] Giovanella L, Treglia G, Iakovou I, et al. 2020 EANM practice guideline for PET/CT imaging in medullary thyroid carcinoma. Eur J Nucl Med Mol Imaging 47 61–77. (10.1007/S00259-019-04458-6) 31482429

[bib5] Giovanella L, Garo ML, Ceriani L, et al. 2021 Procalcitonin as an alternative tumor marker of medullary thyroid carcinoma. J Clin Endocrinol Metab 106 3634–3643. (10.1210/CLINEM/DGAB564) 34382653

[bib6] Giovanella L, Deandreis D, Vrachimis A, et al. 2022 Molecular imaging and theragnostics of thyroid cancers. Cancers 14 1272. (10.3390/CANCERS14051272) 35267580 PMC8909041

[bib7] Hayes AR, Crawford A, Al RK, et al. 2021 Metastatic medullary thyroid cancer: the role of 68Gallium-DOTA-somatostatin analogue PET/CT and peptide receptor radionuclide therapy. J Clin Endocrinol Metab 106 E4903–E4916. (10.1210/CLINEM/DGAB588) 34379772

[bib8] Jara MA & Castroneves LA 2025 Overview of management and therapeutic advances in medullary thyroid cancer. Endocr Oncol 5 e240077. (10.1530/EO-24-0077) 40084047 PMC11906152

[bib9] Niederle MB, Scheuba C, Riss P, et al. 2020 Early diagnosis of medullary thyroid cancer: are calcitonin stimulation tests still indicated in the era of highly sensitive calcitonin immunoassays? Thyroid 30 974–984. (10.1089/THY.2019.0785) 32056502

[bib10] Wells SA, Asa SL, Dralle H, et al. 2015 Revised American Thyroid Association Guidelines for the management of medullary thyroid carcinoma. Thyroid 25 567–610. (10.1089/THY.2014.0335) 25810047 PMC4490627

